# Heterotopic Ossification of the Vascular Pedicle after Maxillofacial Reconstructive Surgery Using Fibular Free Flap: Introducing New Classification and Retrospective Analysis

**DOI:** 10.3390/jcm10010109

**Published:** 2020-12-30

**Authors:** Michael Knitschke, Kelly Siu, Christina Bäcker, Sameh Attia, Hans-Peter Howaldt, Sebastian Böttger

**Affiliations:** Department of Oral and Maxillofacial Surgery, Justus-Liebig-University, Klinikstrasse 33, 35392 Giessen, Germany; kelly.siu@dentist.med.uni-giessen.de (K.S.); christina.baecker-2@dentist.med.uni-giessen.de (C.B.); Sameh.Attia@dentist.med.uni-giessen.de (S.A.); HP.Howaldt@uniklinikum-giessen.de (H.-P.H.); Sebastian.Boettger@uniklinikum-giessen.de (S.B.)

**Keywords:** reconstructive surgery, microsurgery, fibular free flap, FFF, heterotopic ossification

## Abstract

Heterotopic ossification (HO) is one of the described phenomena after maxillofacial reconstructive surgery using fibular free flap (FFF) at the reception-site. The aim of this study was to determine the radiological incidence and form of HO along the fibular vascular pedicle as well as the rate of clinical symptoms if present. CT-scans of 102 patients who underwent jaw reconstructive surgery by using FFF from January 2005 to December 2019 were evaluated concerning the presence of HO. Subsequently, the patient files were evaluated to identify the cases with clinical signs and complications related to the presence of HO. A radiological classification of four different HO types was developed. Out of 102 patients, 29 (28.43%) presented radiological findings of HO. Clinical symptoms were recorded in 10 cases (9.8%) (dysphagia (*n* = 5), trismus (*n* = 3), bony masses (*n* = 2)) and from these only five (4.9%) needed surgical removal of calcified structures. HO occurs significantly in younger patients (mean 52.3 year). In maxillary reconstructions, HO was radiologically visible six months earlier than after mandibular reconstruction. Furthermore, HO is observed after every third maxilla and every fourth mandible reconstruction. This study developed for the first time a classification of four distinct HO patterns. HO types 1 and 2 were mostly observed after mandible reconstruction and type 4 predominantly after maxilla reconstruction.

## 1. Introduction

The fibular free flap (FFF) is the workhorse of defect-oriented reconstruction after combined hard and soft tissue resections within the maxillofacial region [1]. The graft was firstly introduced by Hidalgo et al. [2] and quickly considered as a safe and reliable osteo-fascio-cutaneous graft which is widely used worldwide. The available bone length is usually sufficient for reconstruction of the lower jaw up to class IV [3], offering the possibility of satisfactory oral rehabilitation using endosseous dental implants [4,5,6]. FFF provides a vascular pedicle of sufficient length for use in the entire head and neck region between forehead and clavicula [7] and shows an overall low donor site morbidity [8]. Furthermore, there is the option of forming one or more septo-cutaneous skin paddles, which are suitable for flap monitoring as well as for closing soft tissue defects of the head and neck region. Initially, a stable and sufficient vascular supply of the septo-cutaneous skin paddle was doubted [9], but as this graft became more widespread, knowledge of vascular supply via the perforator vessels of the septum intermusculare posterius and perforator vessels around the musculus soleus was improved [10,11,12].

There is still debate about the reconstruction time point, and whether it is best to perform reconstruction immediately or at a delayed time after the ablative oncological procedure. The necessity of cervical lymphadenectomy offers an ideal approach to suitable vessels for micro-anastomosis. Therefore the time of oncologic resection is the best time for reconstruction [13]. Another advantage is a decrease in surgical procedures and the opportunity for oral rehabilitation in less time [14]. Improved morphological assessment techniques such as frozen-section analysis and flat-panel volume computed tomography of the removed tissues allows an assessment regarding the complete resection of the tumor [15]. Such extended defects are often complex. Combined soft and hard tissue defects after surgery require free vascularized tissue transplant [16]. Wound healing at soft and hard tissue starts immediately after transfer of a combined osteo-fascio-cutaneous FFF. Fracture healing between the original jaw and fibular bone is a highly complex process. Several molecular interactions and gene regulatory processes are necessary for physiological bone growth. Bone healing and regeneration, as with wound healing of any other tissue, follow a sequential process with hematoma formation, tissue inflammation, and recruitment of stem cells. Finally, angiogenesis and bone remodeling will be initiated and continued [17]. Often described complications after reconstruction of maxillofacial structures are delayed wound healing and infection at both donor and receptor-site, (sub-)total flap loss [8,18], plate-related complications (infection, loosing of screw), plate exposure, and osseous non-union [19,20]. Additionally, function problems such as dysphagia, speech complaints, bulky skin paddle, reduced mouth opening, and scars were recorded [18]. Another reason for complaints and functional impairment at the reconstructed area is so-called heterotopic ossification (HO) [21,22].

HO is defined as mature lamellar bone tissue in extra-skeletal soft tissues [23]. Regardless of its genesis, HO has an endochondral structure, which lays on a cartilaginous matrix [24]. Concerning free flaps, HO of the vascular pedicle is described in the literature as a known complication of FFF [25,26,27,28]. Remarkably, evaluation of CT-scans shows frequencies up to 65% [22,29,30]. In detail, there is a broad discrepancy between radiological presence and clinical symptoms of HO. Until now, no literature has been published on the role of virtual planning, necessary patient specific cutting guides for transplant shaping, or the onset of HO. Different theories of origin are discussed, considering the periosteal tissue of the vascular pedicle [26] as well as local mechanic factors and cytokine interactions as the keys role [31]. There is some evidence that FFF harvesting technique and remaining periosteum at the vascular pedicle play a relevant role in HO formation [21,32]. Interestingly HO is also reported in non-osseus transplants like fascio-cutaneous radial forearm free flaps and septomyo-cutaneous lateral upper arm flap, all without any contact to, or included, periosteum [33,34].

The aims of this study are:

1. to estimate the radiological and clinical form and frequency of HO,
2. to define and compare different radio-morphological HO types introducing a new classification,
3. to report the surgical intervention rate for removing calcified structures,
4. and to investigate whether there is a correlation between: analog vs. digital planning, reconstruction methods (immediate vs. delayed), fibular segments and the occurrence of HO.

## 2. Material and Methods

### 2.1. Study Design and Patient Population

The study was conducted as a monocentric, retrospective study. CT scans and cone beam CTs (CBCT) of patients who underwent successful FFF in the head and neck region from January 2005 to December 2019 were analyzed concerning the presence of heterotopic ossification (HO). The evaluated CT-scans were initiated either within the course of radiation planning or routine follow-up examination. CBCT were mostly performed to plan the insertion of dental implants for oral rehabilitation.

### 2.2. Study Parameters and Evaluator Calibration

The following parameters were collected: age at flap transfer, sex, primary diagnosis, planning procedure, location and type of defect, number of used fibula segments, extension of neck dissection, irradiation, clinical symptoms of HO and need for surgical intervention if HO was clinically symptomatic. All CT and CBCT-scans were analyzed independently for presence of HO by two authors (KS and MK). If they disagreed, it was planned to take a third author’s opinion (SB) in consideration, which was not necessary. MK and SB are experienced maxillofacial surgeons. KS is a fifth year dental medicine student.

### 2.3. Inclusion and Exclusion Criteria for Study Subjects

All patients who underwent a successful reconstruction of the maxilla or mandible (simultaneous or two-staged) with a FFF were enrolled in this study. Inclusion criteria were the presence of at least two CT-scans of the region of interest. Only CT-scans with a slice thickness ≤5 mm were included to ensure optimal HO detection. The minimum follow-up interval was four months. Cases with incomplete data sets, patient records, and those with less than two postoperative CT- or CBCT-scans of the head and neck region or a CT layer thickness >5 mm were excluded.

### 2.4. A New Classification of HO of the Vascular Pedicle and Periosseous Tissue Based on Radiological and Clinical Follow-Up

**Figure 1:**
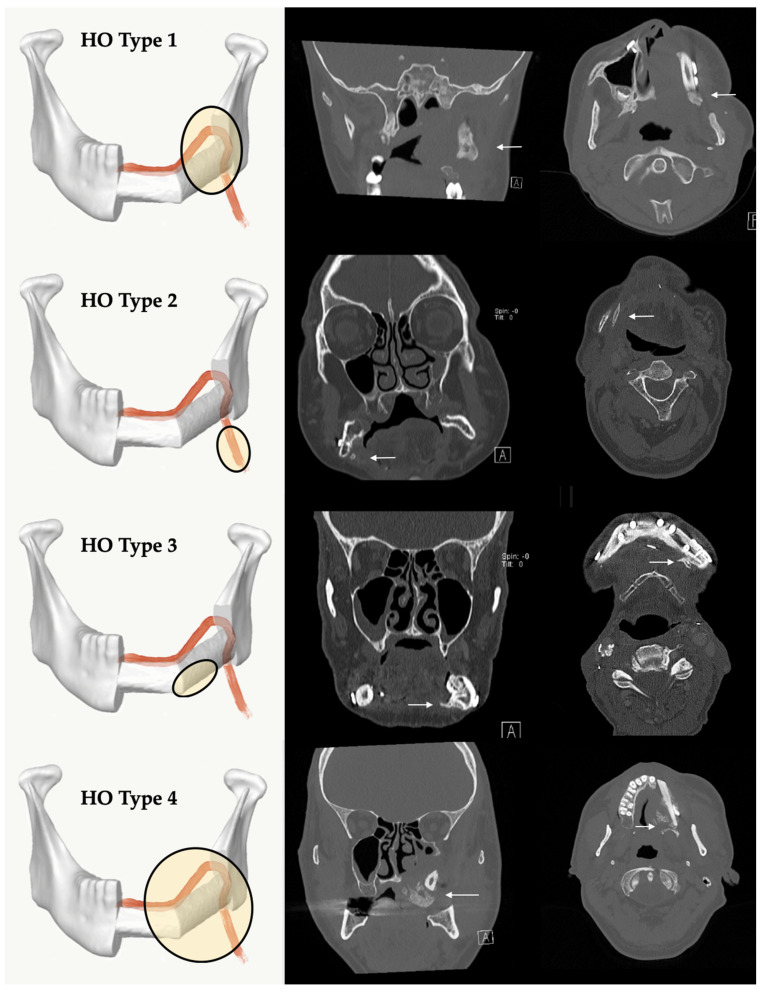
Classification of different radiological types of heterotopic ossification (HO): Type 1 = HO at the transition zone from fibula graft to vascular pedicle, Type 2 = HO isolated at the pedicle without contact to the fibula, Type 3 = HO appears isolated at the periosseous tissue without involvement of the vascular pedicle, Type 4 = a combination with ossification of the pedicle and periosseous tissue. The white arrows mark region of interest. A = Anterior.

**Figure 2:**
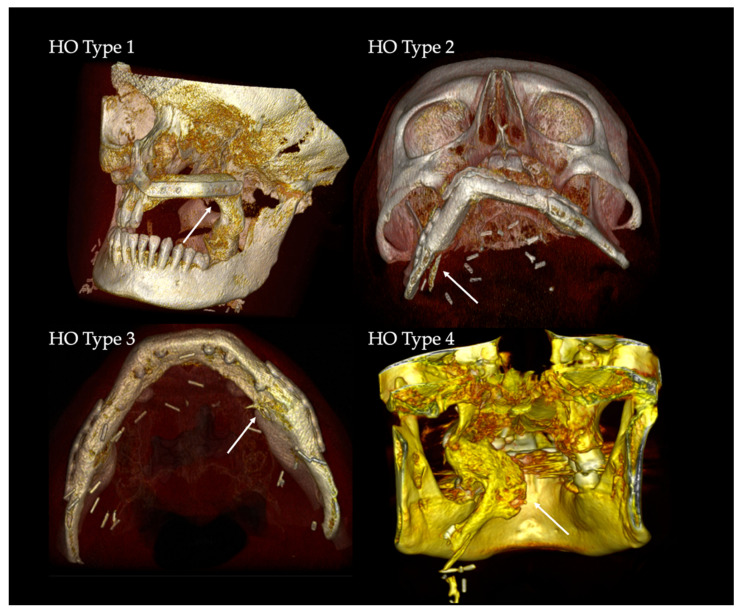
3D-volume rendering of clinical examples of HO Types 1–4. The white arrows mark the regions of interest.

Systematic evaluation of the patient records was performed to identify the cases in which the clinical presence of HO in the vascular pedicle region (palpable submandibular “bone-hard” swelling, ongoing difficulty swallowing and/or the periosteal tissue) led to clinical complications. For the radiological description, five types of HO patterns were defined (Figure 1 and Figure 2): no recognizable ossification of the vascular pedicle or perosseous tissue corresponds to type 0. Type 1 shows an ossification at the transition zone from fibula graft to vascular pedicle. Type 2 shows isolated ossification of the vascular pedicle without contact to the fibula. Type 3 is defined as an isolated HO of periosseous tissue without pedicle-associated ossification. Type 4 is a combination of HO of the vascular bundle and periosseous tissue.

### 2.5. Statistical Analyses

Chi-square-test was used to compare the frequency of HO in males and females. Students *t*-test was performed to compare the mean age at FFF-transfer between groups with and without radiological sign of HO, after verification of normality. *p* < 0.05 was defined as statistically significant. The statistical analysis (analogous to Kaplan-Meier survival function) was carried out with SPSS 25 (SPSS Inc., Chicago, IL, USA, http://www.spss.com).

### 2.6. Ethics Statement/Confirmation of Patients’ Permission

The study was approved by the local Ethics Committee of Justus-Liebig University Giessen (AZ34/20) and patients’ permission/consent was not necessary in this retrospective study. The patients consented that their intraoral pictures and X-ray images could be used anonymously in the publication. In addition, all data in the Microsoft Excel spreadsheet were pseudonymized.

## 3. Results

A total of 149 cases fulfilled the defined inclusion and 47 cases matched the exclusion criteria. A total of 102 FFF cases (M: *n* = 66 (64.7%), F: *n* = 36 (35.3%)) with complete data records during the period January 2005 to December 2019 were analyzed in the study (Table 1). The average age at surgery time in the HO- group was 58.69 ± 11.92 year (median 59.91 years, range 32.58–82.75 years) (follow-up 45.16 ± 44.49 months) and in the HO+ group 52.30 ± 14.39 years (median 53.92 years, range 14.75–76.83 years) (follow-up 66.59 ± 45.04 months). Both groups were tested for normal distribution. There was a significant age difference between the HO+ and HO- group (*p* = 0.0236). FFF was used for maxillary reconstruction in 26 cases and for mandibular reconstruction in 76 cases.

Table S1 in the supplementary material depicts the collected data of our 29 patients with radiological HO (M: *n* = 25 (86.2%), F: *n* = 4 (13.8%)). Extraosseous ossification occurred in 34.6% (*n* = 9 out of 26) after maxillary and in 26.3% (*n* = 20 out of 76) after mandibular reconstruction. The difference was not statistically significant (*p* = 0.4553).

**Figure 3:**
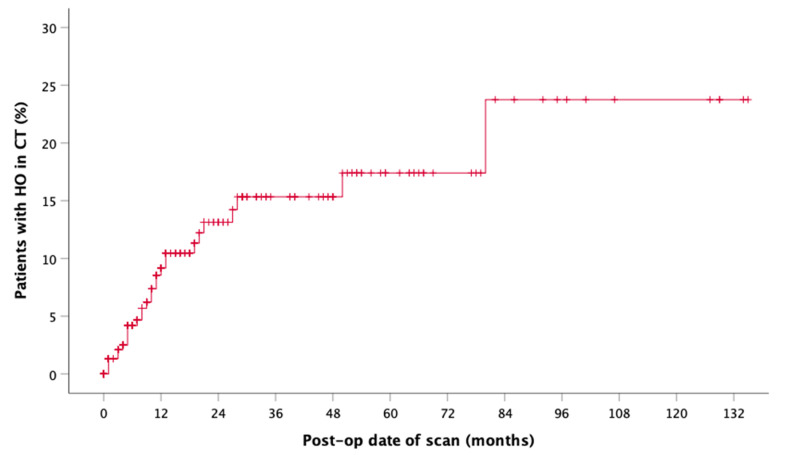
Incidence function shows the overall radiographic presence of HO aligned to the postoperative date of CT (*n* = 29).

**Figure 4:**
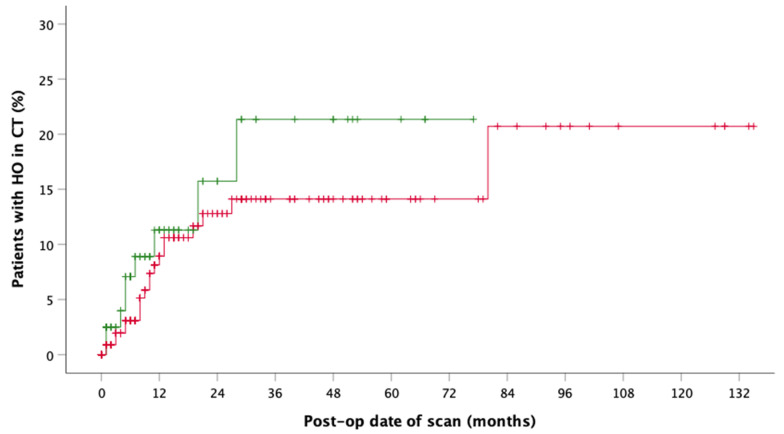
Incidence function shows the radiographic presence of HO by postoperative date of CT in maxillary (green, *n* = 9) and mandible (red, *n* = 20) reconstruction.

The onset of HO in the CT-scan was drawn as incidence function (Kaplan Meier function), cumulative for reconstruction of maxilla and mandible with FFF (Figure 3). After an average time of 13.48 months (median 9.0 months), HO was observed in CT scans. Comparing time of detection of HO after maxillary (median = 5 months, 95% CI = 4.0–20.0 months) and mandibular reconstruction, it is noticeable that maxillary HO occurred six months earlier than mandibular HO (median = 9.5 months, 95% CI = 5.0–13.0 months) (Figure 4). Incidence functions for reconstruction of continuity of mandible and maxilla were calculated separately (Figure 4).

There were no statistically significant differences in the presence of radiological HO with regards to time of reconstruction (*p* = 0.1128) and the planning procedure, which was either analog (hand-bent, freehand osteotomized) or virtual (CAD/CAM plate, CAD/CAM cutting guides) (*p* = 0.3806). Jaw reconstruction was necessary in case of malignant disease in 89.22% of cases (*n* = 91) and 22 out of 91 developed HO. Reconstructions of continuity of the maxilla and mandible were classified by site and extent of the defect (Table 1). The extent of cervical lymphonodectomy (selective vs. modified radical neck dissection) and no lymphonodectomy showed no influence on the presence of HO.

After adjuvant radiotherapy (RT) with <60 Gy, however, a near to similar incidence (*n* = 10 out of 30 (33.3%)) was observed compared to no RT (*n* = 16 of 47 (34.0%)). Within the subgroup of total irradiation dose of ≥60 Gy, two cases of HO (*n* = 2 of 22 (9.1%)) were documented. These observations were not statistically significant.

**Figure 5:**
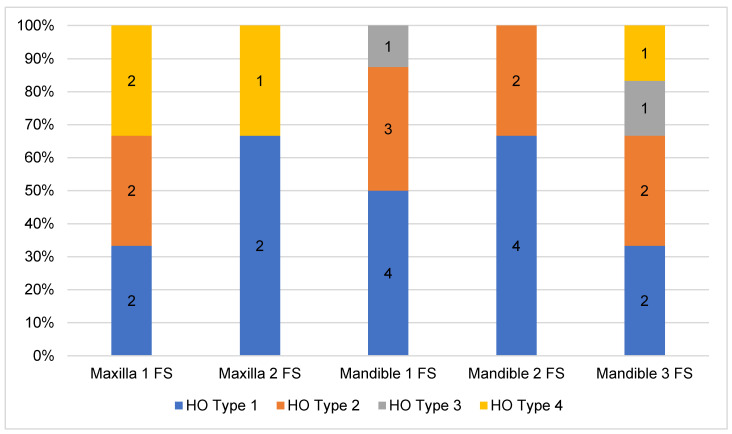
Distribution of HO types according to the number of used fibula segments for jaw reconstruction.

**Figure 6:**
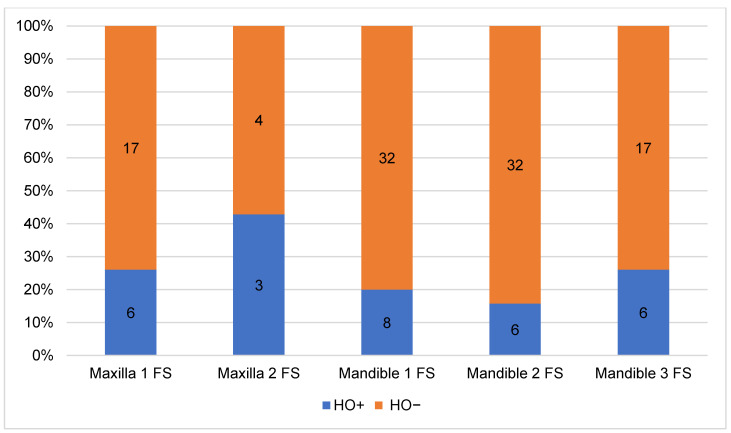
Relative proportion of HO in relation to localization and number of fibula segments (FS) used for jaw reconstruction.

The analysis of the CT-scans showed four different patterns (types) of HO. Distribution and results are shown in Table 2. HO Type 1 was most frequently observed in four cases after maxilla (13.79%) and in 10 cases after mandible (34.48%) reconstruction with FFF. Isolated ossification of the pedicle (Type 2) accounted for seven cases in the mandible (24.14%) and 6.9% (*n* = 2) in the maxilla. In three cases, combined ossification (Type 4) after maxilla reconstruction (10.34%) and only one case after mandibular reconstruction were recorded. HO Type 3 was only recorded in two cases after mandibular reconstruction (6.9%). Figure 5 illustrates the distribution of HO types according to the number of fibula segments used. A homogeneous distribution of HO Type 1 is shown across all reconstruction localizations and shapes, while Type 2 and Type 4 are found in both types of jaw reconstruction. Type 3 could only be observed after mandibular reconstruction. Figure 6 outlines the appearance of HO in CT scans considering the number of used fibula segments in maxillary or mandible reconstruction. HO occurred more frequently after bi-segmental (33.3%) maxillary reconstruction compared to mono-segmental (29.1%) maxillary reconstruction. In the mandible, HO was most frequently observed after tri-segmental (26%) reconstruction compared to mono-segmental (20%) and bi-segmental (15%) reconstruction.

T-test showed a significant difference for the parameter ‘age’ and presence of HO (*p* = 0.0236) disregarding reconstructed upper or lower jaw. A statistical sub-analysis of the parameter ‘age’ concerning occurrence of HO in maxillary (*p* = 0.158) or mandible (*p* = 0.232) reconstruction revealed no statistically significant difference.

**Figure 7:**
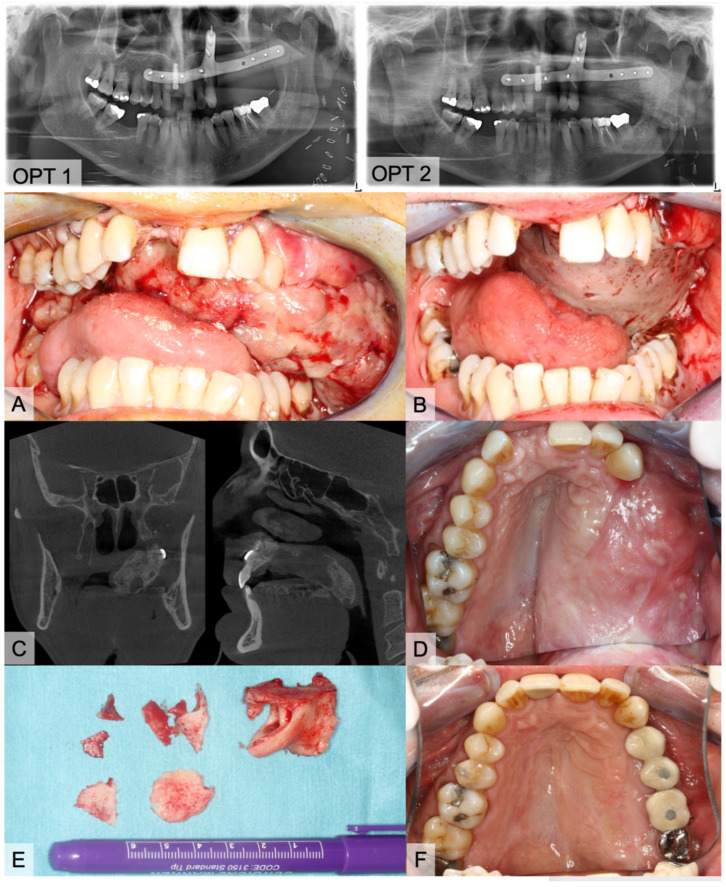
Images of an 54-year-old, healthy male who underwent subtotal hemi-maxillectomy due to a second recurrence of an odontogenic kerato-cyst (OKC). After more than one year later, we performed a virtually planned mono-segmental FFF transfer without a skin paddle for maxillary reconstruction (OPT 1). Three days after discharge, he consulted the emergency unit with progressive dyspnea and fear of asphyxiation in dorsal position. Clinical inspection showed large masses of soft and vulnerable granulation tissue (**A**). After carefully removing the tissue, we covered the bone with a split skin graft from the thigh (**B**). Six months later, we planned implantological rehabilitation. OPT and CBCT were accomplished and presented large bone masses at the palate (**C**, OPT 2). Clinical impression is shown on image (**D**). Prior to the insertion of four dental implants, we molded the FFF and removed bone masses (**E**). Implants were exposed four months later. Figure (**F**) shows the final prosthetic rehabilitation.

The evaluated medical records of the HO+ group showed clinical symptoms in 10 cases with swallowing difficulties (*n* = 5), trismus (*n* = 3), and palpable bony masses in level I (*n* = 2). Clinical complaints occurred after an average time of 6.3 months. Surgical intervention with a removal of calcification was only necessary in five cases. One of the patients (Figure 7) had a two-staged, virtually planned mono-segmental maxillary reconstruction after previous resection of a recurrence of a odontogenic kerato-cyst (OKC). Early after flap transfer there was an extensive overgrowth of granulation tissue with subsequent periosteal ossification in the area of the palate and parts of the vascular pedicle (HO type 4). He suffered from severe dyspnea and dysphagia, prompting the surgical removal of the granulation tissue and parts of the heterotopic ossification. As a temporary wound closure, the defect was covered with a split skin graft and a screw-fixed dressing plate for five days. With further progress, a modelling osteotomy was performed simultaneously with the insertion of dental implants. Histopathologic evaluation of the tissue specimen confirmed the presence of mature bone tissue (Figure 7).

## 4. Discussion

### 4.1. HO after FFF in Literature

Radiologically detected ossifications of the vascular pedicle of osteo-fascio-cutaneous FFF are described in the literature with a high incidence of up to 65% [22,29,30]. Table 3 lists publications that reported the radiological and clinical incidences of HO after FFF. Individual case reports on the occurrence of HO after radialis flap [34], lateral upper arm flap [33], and scapula flap [36] have been published. HO rarely seems to lead to clinical symptoms and surgical correction is required even less frequently.

Different theories on the origin of HO are discussed. In osteo-fascio-cutaneous flaps, periosteal tissue near to the vascular pedicle seems to play a central role [26] as well as local mechanical factors and cytokine interactions [31]. The FFF harvesting technique with remaining periosteum on the vascular pedicle has significant influence on the development of HO [21,32]. All FFF in the investigation were harvested by the lateral approach [40] on the angled, perfused lower leg. In case of multi-segmental reconstructions, the corresponding preparation of the fibula was completed with the application of miter cuts (freestyle or prefabricated cutting guides) and osteosynthesis (hand-bent or custom-made). The proximal, free fibula segment was de-periosted, osteotomized and either discarded or used as a free graft (e.g., particulate). The exposed periosteum partially remains on the vascular pedicle, which is surrounded by a muscle layer. Preferred microvascular recipient vessels were the A. thyroidea superior or A. facialis. Direct end-to-end anastomosis of the external carotid artery was rarely used. While a long vascular pedicle facilitates microsurgical-vascular handling, it increases the risk of kinking pedicle. The longer vascular pedicle is prepared to a larger extent than the area with remaining adherent periosteum will be, from which HO can commence. Especially in young patients, careful periosteum-free vascular pedicle preparation should therefore be emphasized [39]. Tarsitano et al. could not observe any case of HO [36] by this modified harvesting technique (*n* = 20) with removal of the periosteum adhering to the vascular pedicle, which emphasizes a possible key role of osseo-inductive cells of the periosteum in the etiology of HO [21]. The periosteum is suspected to be a privileged target of recombinant human bone morphogenetic protein 2 [36]. Colletti et al. conclude that careful separation of the vascular pedicle from the periosteum seems to prevent HO [38]. An additional modification of this FFF harvesting procedure described by Kim et al. [32] suggests that, due to virtual planning, the extent of required fibula is exactly known and thus supra-periosteal proximal preparation can be performed after branching out of the fibular artery to the planned proximal osteotomy. They state that it is possible to omit one surgical step and thus shorten flap harvesting time [32]. Despite supra-periosteal dissection, there are cases in which pedicle ossification occurs [41]. The supra-periosteal dissection was not performed in the investigated group. 

In addition to the outlined role of periosteal osteoprogenitor cells, other theories are discussed. The theory of fracture repair focuses on the presence of all cellular and molecular players such as bone morphogenetic protein (BMP), which are important for bone healing [42]. The ligands of the BMP signaling pathway, BMP-2 and BMP-4, have been known to induce bone formation for several decades [43,44]. Multiple studies have used this fact to recreate HO in vivo [45,46,47] or analyze the mechanistic basis of HO in vitro [48,49].

Blood flow and perfusion theory aims at changed pressure conditions in the arterial and venous system of the FFF after transfer of the arterial moderate and relative venous high pressure situation (valves, muscle pump) on the lower leg compared to the high arterial and low venous pressure situation of the head and neck region [36]. In addition, after microsurgical anastomosis of the vessels, there were changes in the flap perfusion. After flap dissection small arterial and venous collaterals were interrupted. Both factors, a higher arterial perfusion pressure at the cervical acceptor site and less opportunity of flap drainage to the surrounded tissue, can inflate the flap tissue. The mechanical theory describes the influence of physiological forces due to micro-stress on the callus and the bone healing of the attachment surfaces in the upper and lower jaw [21,25,31,36].

### 4.2. Classification of Four Different HO Patterns and Biological Etiology

The HO classification suggested in this study distinguishes four types of HO. Type 1 is the most frequently observed variant across all reconstruction sites and forms. Concerning the referred theories of HO etiology, the bone healing/fracture repair theory describes the origin of HO from the resection site. Functional stress to the stabilized bone graft induces micromotion and enhances bone healing and callus growth [36]. We hypothesize that, as a result, the calcified tissue is a type of excessive bone healing that originates from the resection site and courses along the remaining periosteum at the pedicle by molecular stimulation from osteoprogenitor recruitment [31,42,50].

Type 2 may be the result of isolated periosteal cells in contact with osteocytes along the pedicle from osteo-cutaneous FFF [26,51,52,53]. A long vascular pedicle with remaining periosteal cells therefore offers more potential for the development of HO [25]. We expected that HO Type 2 will be more common, especially in maxillary reconstructions but we found only two cases after maxilla, but seven cases after mandible reconstruction. However, molecular interactions and the influence of BMPs on the induction of wound healing could promote HO formation [31]. Local factors could have additional influence after bone resections.

HO Type 3 was only observed after mandible reconstruction. Its clinical appearance is similar to torus mandibularis. The occurrence of ectopic oral bone formation is probably the result of functional aspects of mastication [54,55]. Thus, this HO type could be triggered by manipulation of the periosteum and its dissection during preparation and maintained by functional factors induced by mastication. In addition, mechanical stress (tension) supports BMP signaling [31]. Keeping this in mind, we expected to find HO Type 3 occurring more frequently in poly-segmental reconstructions close to the resection sites and more particularly at intersegmental graft sites. There are discrepancies between cross sections of resection sites and FFF. Remnant free periosteum after poly-segmental shaping procedure around vascular bundle needs to be considered as an origin of HO. There were no clear reasons why HO Type 3 was not found after maxillary reconstruction. One thinkable hypothesis is that HO Type 3 origin might be an hyperperfused and inflated bone feeder vessel via the above-mentioned mechanisms of blood flow and perfusion theory. For maxillary reconstruction the strong and rigid tissue of the gum might be a sufficient resistance to prevent its development in compare to the more loose and wide tissue of the floor of the mouth.

Changes in blood flow and pressure could also induce HO Type 4 [36]. Three cases after maxilla and one after mandible reconstruction can be assigned to HO Type 4. This pattern was observed in younger patients (mean age 43.58 y). Focused on the morphological architecture of the surrounding soft tissue, the micro-vessels seem to be inflated. There is also an increase in bone and blood vessels during callus healing normally reported in stabilized fractures [56]. In the clinical case presented in Figure 7, we hypothesize that the overwhelming granulation of the soft tissue might be an early clinical expression of upcoming HO. Only in this case was no skin paddle used to cover the soft tissue of the graft which obviously led to granulation tissue. The low pressure from the surrounding functional soft tissue (tongue, floor of mouth, cheek) appears to be one possible cofactor in the development of HO. In maxilla reconstruction, a tunnel for the vascular pedicle has to be dissected bluntly. Following McCarthy’s remarks, a superficial tunnel in the face-lift plane facilitates the course of vessels and, if the maxillary tubercle is resected, access can be gained by a parapharyngeal approach medial to the mandible [57]. Here, we also recognized HO (Figure 1, Figure 2 and Figure 7). In mandible reconstruction, HO Type 4 was not as distinctive as in maxilla reconstruction. It is conceivable that the neck tissue surrounding the vascular pedicle sufficiently counteracts the changed perfusion pressures after flap transfer. This could be a reason why pronounced changes such as in the tunnel area of the oral cavity after maxilla reconstruction could not be observed.

### 4.3. Impact of Analogous and Virtual Planning

In the literature, HO appears to occur more frequently in maxillary reconstructions [27,36]. HO was found in 34% (*n* = 9 of 26) of all cases of maxillary reconstructions with FFF. This corresponds to a ratio of approximately 1:3. However, overall, more than two thirds of radiological observed HO cases (69%, *n* = 20) were recorded after mandibular reconstruction. At a total number of 76 successfully performed mandibular reconstructions, we identified 20 cases of HO. This corresponds to a ratio of about 1:4. Since 2015, we have been planning jaw reconstructions digitally. The results show a radiological incidence after analogous (11.45%) and digitally (10.69%) planning procedures. However, in the early years of jaw reconstruction, cases were planned analogously and were realized “manually” [58]. In recent years computer-assisted planning is clinical routine and, in addition to custom-made resection and cutting guides, patient-specific manufactured osteosynthesis plates are available [59,60,61,62,63,64]. The selection of the donor region and reconstruction morphology is the subject of current research into the use of algorithm-based automated procedures [65]. There was no significant difference in the presence of HO between virtually planned and CAD/CAM stabilized fibula (10.69%) grafts than in those of analog and hand-bent osteosynthesis (11.45%).

### 4.4. Frequency of Clinical Symptoms and Surgical Removal of HO

Although the ossification of the vascular pedicle according to radiological criteria seems to be common [29], clinical complications seems to be rare. Despite a radiological incidence of over 28.43%, we identified 10 cases of clinically symptomatic HO (9.8%) in the investigated collective with swallowing difficulties (*n* = 5), trismus (*n* = 3), and bony masses in level I (*n* = 2). Only five patients (4.9%) needed a surgical intervention to remove calcification. On average, clinical complaints occurred after 4.4 months and surgical removal of calcified structures was performed after 12.6 months. HO and the planned removal of HO-related structures is a definite indication for surgery. In our opinion, changing the surgical harvesting procedure (supra-periosteal preparation of the proximal pedicle) does not seem to be advisable regarding the background of the low incidence of clinical symptomatic HO and the multifactorial genesis, where the periosteum plays an essential role.

However, excessive new bone formation in the entire graft area (HO Type 4) can result in massive clinical impairment [27]. Depending on graft location, the course of the vascular bundle and the region of anastomosis, the incidence of HO may be underestimated. According to the literature, HO is rarely symptomatic in a way that surgical intervention is indicated. However, removal of ectopic bone mass may be necessary in cases of impaired movement of mandibula, pain during mastication or disturbing submandibular masses in neck level I or II after exclusion of a recurrence of the proper oncological disease [21,36]. It is remarkable that all five surgically treated cases in the present investigation occurred after maxillary reconstruction, because of functional impairment (Table 2). In case of extensive periosseous HO, modelling osteotomy for recontouring and application of a screw-fixed dressing plate may be considered as a therapeutic strategy. Ossification of the vascular pedicle at the transition from FFF to pedicle (Type 1) and isolated HO of the pedicle (Type 2) can be treated surgically if clinical symptoms are clear. Careful, subtle preparation of vascular stalk is only necessary during the early phase after transplantation until the graft is adequately perfused with blood. Myon et al. report that, in one case, the removal of a pedicle-associated HO did not lead to any reduction of the flap vitality [36]. Sufficient neovascularization to allow FFF survival independent of the vascular pedicle has been reported to occur within 4 to 12 weeks [66].

### 4.5. Effect of Irradiation on HO Occurrence

It is interesting to note that adjuvant irradiation (group <60 Gy and ≥60 Gy) leads to a decrease of the number of HO cases (not statistically significant). In orthopedic surgery, the hip is the most common site of HO development and treatment. Radiotherapy (RT) has been shown to be particularly effective in the hip, knee, and elbow area. In studies, uni-fractionated radiation application of 7 Gy was performed after elbow surgery and was associated with favorable functional and radiological result [67]. Another study of nine patients with clinically significant HO at the elbow reported a majority of clinical improvement after irradiation. In this study, 5 Gy in two fractions and 6–7 Gy in one fraction were applied. The mean follow-up was 7.7 months [68]. For prevention of HO after hip endo-prosthetics, prophylactic radiotherapy with 1 × 7 Gy immediately before or up to 24 h after surgery is recommended [69]. This leads to the question of the influence of fractionation at overall low doses of 7 to 16–17 Gy. In adjuvant radiotherapy in the head and neck region single applications between 1.8–2.2 Gy are normally used. In summary, there are four main differences in oncological adjuvant RT in head and neck: low single application dosage between 1.8–2.2 Gy per fraction, onset of RT up to four weeks after operation, overall cumulative irradiation dose (56–72 Gy), and amount of 33–34 fractions.

### 4.6. Limitations of This Study

The evaluation shows statistically significant differences concerning ‘Age’ and the occurrence of HO. However, subgroup analysis concerning maxillary or mandible reconstruction mean age was without any significant results. However, where there is a difference in mean of 10 years between the HO+ and HO− groups in maxilla reconstructions, the number of cases is too small and the age distribution too broad to reach statistical significance.

Limitations of the presented study are the retrospective design and the lack of defined imaging time points, so that the mean or median first observation time of HO at 3.48 and 9.0 months appears late. Glastonbury et al. described typical imaging findings of HO in three cases only one month after FFF [29]. However, the incidence functions show that about half of the observed cases become visible in CT scan within the first 12 postoperative months. The second half only develop in the following six years. The reasons for this remain unclear. Bone or fracture healing is complete so that continuous remodeling processes could play a role. Our mixed patient collective was mostly formed of oncologic patients (87.78%). Two third were male and one third female. About 86% of the radiologically detected HO cases were male patients which could be a selection bias. Therefore, the bias continued in the clinical (M = 8, F = 2) and in the surgically treated group (M = 4, F = 1).

Finally, further investigation and more data is necessary to validate this newly introduced classification. Any new classification in medicine should be verified by an independent cohort.

## 5. Conclusions

Extraosseous heterotopic ossification (HO) is a known phenomenon of osteo-cutaneous fibular free flap (FFF) after ablative tumor surgery and jaw reconstruction and can lead to clinical complications. Trismus, mastication pain and/or rough submandibular masses could be an indicator of HO if postoperative scars and tumor recurrence are excluded.

Radiologically, HO appears to occur more frequently in younger and male patients. No correlation between HO and the method of planning (analog vs. digital), the type of osteosynthesis (hand-bent vs. custom-made), or time of reconstruction (immediately vs. delayed) were found. A modified harvesting technique with a vascular pedicle without periosteal tissue seems to be an effective method to avoid heterotopic ossifications. This method could prevent the development of HO in maxillary reconstruction. In case of extensive HO, the modelling osteotomy for jaw recontouring and the application of a screw-fixed dressing plate could be considered as a therapeutic strategy.

To our knowledge, this is the first study to describe a classification of four different radiological HO patterns. Further studies for the classification’s validation are required.

## Figures and Tables

**Table 1:** Clinical details of 102 patients after reconstruction of continuity defects of maxilla and mandible with fibular free flaps.

*n* = 102	HO− *n* = 73 (71.57%)	HO+ *n* = 29 (28.43%)	
Age (year), SD	58.69 ± 11.92	52.30 ± 14.39	*p* = 0.0236
Follow-up (months), SD	45.16 ± 44.49	66.59 ± 45.04	
HO duration until observation (months)		mean 13.48, median 9 ± 16.54	
Surgical intervention		5	
Sex			
Male	41 (40.20%)	25 (24.51%)	
Female	32 (31.37%)	4 (3.92%)	*p* = 0.5096
Preoperative planning/osteosynthesis			
Analog/hand-bent	45 (44.12%)	15 (14.71%)	
Virtual/custom-made	28 (27.45%)	14 (13.73%)	*p* = 0.3806
Reconstruction			
Immediately	60 (58.82%)	19 (18.63%)	
Delayed	13 (12.75%)	10 (9.80%)	*p*= 0.1128
Diagnosis			
Malignant	69 (67.65%)	22 (21.57%)	
Benign	4 (3.92%)	4 (3.92%)	
Other (ORN, BPONJ, OM)		3 (2.94%)	
			
Location			
Maxilla	17 (16.67%)	9 (8.82%)	
Mandibula	56 (54.90%)	20 (19.61%)	*p* = 0.4553
Maxilla defect type (Brown et Shaw 2010) [35]			
II	15 (57.69%)	8 (30.77%)	
III	2 (7.69%)	1 (3.85%)	
Mandible defect type (Brown et al. 2016) [3]			
I	15 (19.74%)	7 (9.21%)	
Ic	4 (5.26%)		
II	13 (17.11%)	3 (3.95%)	
IIc	1 (1.32%)	1 (1.32%)	
III	22 (28.95%)	7 (9.21%)	
IV	1 (1.32%)	2 (2.63%)	
Reconstruction			
Maxilla 1 FS	14 (53.85%)	7 (26.92%)	
Maxilla 2 FS	3 (11.54%)	2 (7.69%)	
Mandibula 1 FS	24 (31.58%)	8 (10.53%)	
Mandibula 2 FS	19 (25.00%)	6 (7.89%)	
Mandibula 3 FS	13 (17.11%)	6 (7.89%)	
Neck dissection (ND)			
None	14 (13.73%)	12 (11.76%)	
Selective ND	23 (22.55%)	4 (3.92%)	
MR ND	36 (35.29%)	13 (12.75%)	n.s.
Neck surgery			
One side	54 (52.94%)	23 (22.55%)	
Both sides	19 (18.63%)	6 (5.66%)	*p* = 0.6213
Postoperative irradiation			
None	31 (30.39%)	16 (15.69%)	
< 60 Gy	20 (19.61%)	10 (9.80%)	
≥ 60 Gy	20 (19.61%)	2 (1.96%)	
Dosage unknown	2 (1.96%)	1 (0.98%)	n.s.

n.s. = not significant; ORN, Osteoradionecrosis; BPONJ, Bisphosphonate related osteonecrosis of the jaw; FS, number of used fibula segments; OM, Osteomyelitis; selective ND summarizes submandibular or supraomohyoidal neck dissection; MRND, modified radical ND.

**Table 2:** HO was classified into four distinct radiological identifiable patterns. The distribution of HO types according to site of appearance (maxilla or mandible) and mean age (Minimum/Maximum), clinical impairments, and necessary surgical interventions are shown (Max., Maxilla; Mand., Mandible).

HO Type	Maxilla		Mandible		Cumulative		Complaints		Surgery	
	*n*	Age (Year) (Min/Max)	*n*	Age (Year) (Min/Max)	*n*	Age (Year) ± SD	Max.	Mand.	Max.	Mand.
0	17	58.65 (32.58/79.08)	56	58.70 (32.83/82.75)	73	58.69 ± 11.92				
1	4 (13.79%)	47.63 (14.75/68.25)	10 (34.48%)	54.59 (37.75/76.83)	14	52.60 ± 15.85	2	1	1	-
2	2 (6.90%)	63.5 (53.92/73.08)	7 (24.14%)	53.81 (30.0/65.66)	9	55.96 ± 13.61	1	2	1	-
3			2 (6.90%)	51.21 (46.08/56.33)	2	51.20 ± 7.25		1		-
4	3 (10.34%)	40.25 (24.41/54.0)	1 (3.45%)	53.58	4	43.58 ± 13.87	3		3	-
	9 (31.03%)	48.69 (14.75/73.08)	20 (68.97%)	53.92 (30.0/76.83)	29	52.30 ± 14.39				

**Table 3:** Heterotopic ossification (HO): overview published cases.

Authors	Flap (*n*)	Incidence on Radiographs	Imaging	Clinical Symptoms/Surgical Removal
Deschler et al., 1997 [37]	FFF (*n* = 38)	No data available	OPT	8% (*n* = 3)
Smith et al., 2003 [27]	FFF, CR		CT	(*n* = 1)
Autelitano et al., 2008 [26]	FFF (*n*~100)	No data available		~4% (*n* = 4)
Gonzales-Garcia et al., 2011 [25]	FFF, CR		CT	(*n* = 1)
Colletti et al., 2014 [38]	FFF (*n* = 92)	No data available		4.3% (*n* = 4)
Acarturk et al., 2011 [39]	FFF, CR		CT	(*n* = 1)
DeConde et al., 2011 [22]	FFF (*n* = 520) *	65% (*n* = 43 out of 66)	CT	2.6% (*n* = 14 out of 520)
Myon et al., 2012 [36]	FFF (*n* = 149) SF (*n* = 13)	9.2% (*n* = 15) (*n* = 14) (*n* = 1)	OPT, CT	3.7% (*n* = 6)
Karagozoglu et al., 2013 [30]	FFF (*n* = 74)	27% (*n* = 20)	OPT	No data
Tarsitano et al., 2013 [21]	FFF sp (*n*= 41) mp (*n* = 20)	17% (*n* = 7) 0%	OPT, CT	4.9% (*n* = 2)
Glastonbury et al., 2014 [29]	FFF (*n* = 32)	50% (*n* = 16)	CT	3.13% (*n* = 1)
Baserga et al., 2016 [28]	FFF (*n* = 68)	No data		4.4% (*n* = 3)
This study	FFF (*n*=102)	28.4% (*n* = 29)	CT, CBCT	9.8% (*n* = 10)/ 4.9% (*n* = 5)

CR Case Report, FFF = fibular free flap, SF = scapula flap, sp = standard procedure, mp = modified supra-periosteal procedure, * from 520 cases only 66 CT-scans were available for multiplanar reconstruction and analysis.

## Data Availability

The data presented in this study are available on request from the corresponding author.

## Supplementary Materials

The following are available online at https://www.mdpi.com/2077-0383/10/1/109/s1, Table S1: Characteristics of HO+ cases (*n*= 29) after FFF.
