# Supplementary material for: Heterotopic Ossification of the Vascular Pedicle after Maxillofacial Reconstructive Surgery Using Fibular Free Flap: Introducing New Classification and Retrospective Analysis
Source: J Clin Med. 2020 Dec 30;10(1):109. doi: 10.3390/jcm10010109 (PMC7794830; doi:10.3390/jcm10010109)
Supplement: Supplementary file 1 [file jcm-10-00109-s001.pdf]

Table S1. Characteristics of HO+ cases (n= 29) after FFF.

ID	Sex	Age at free flap	Primary disease	Resection site	Neck dissection	Defect-type [3, 35]	Fibula-segments	Analog (A) vs. virtual (V) planning	Radiol. onset HO (mo)	HO-type	HO clinical symptoms	Clinical onset HO (mo)	Radiation	Surgical removal
1	M	68.3	OSCC	Max.	None	II	2	A	28	1			-	
2	M	49.7	OSCC	Mand.	MRND ipsi., SOND contra.	I	1	A	1	1			< 60 Gy	
3	M	65.7	OSCC	Mand.	MRND ipsi.	I	1	A	80	2			-	
4	M	57.1	OSCC	Mand.	MRND ipsi.	I	1	A	8	1			< 60 Gy	
5	M	62.4	AMB	Max.	None	II	2	A	11	1	Bony masses	4	-	y (12 mo)
6	M	63.4	OSCC	Mand.	MRND ipsi.	I	1	A	13	2	Swallow-ing	3	< 60 Gy	n
7	M	49.2	OSCC	Mand.	MRND ipsi.	I	1	A	13	1			-	
8	M	47.4	OS	Mand.	SOND ipsi.	III	2	A	27	1			-	
9	M	60.4	OSCC	Mand.	MRND ipsi., SOND contra.	II	2	A	10	1			< 60 Gy	
10	M	46.1	OSCC	Mand.	MRND ipsi.	I	1	A	3	3			-	
11	M	56.3	OSCC	Mand.	MRND ipsi.,	III	3	A	9	3	Swallow-ing	5	< 60 Gy	n

					SOND contra.									
12	F	53.9	ACC	Max.	None	II	1	A	4	2			-	
13	M	42.3	AMB	Max.	None	II	1	V	5	4	Trismus	4	-	y (6 mo)
14	M	45.1	ACIN	Max.	None	II	1	V	20	1			-	
15	M	60.7	OSCC	Mand.	MRND ipsi.	III	2	A	11	2			-	
16	M	37.8	OSCC	Mand.	SOND bilat.	IV	3	V	3	1			-	
17	M	54.0	OKC	Max.	None	II	1	V	7	4	Swallow-ing	1	-	y (9 mo))
18	F	73.1	OSCC	Max.	MRND ipsi.	II	1	V	1	2	Trismus	5	< 60 Gy	y (25 mo)
19	M	53.6	OSCC	Mand.	MRND ipsi, SOND contra.	III	3	V	10	4			-	
20	M	76.8	OSCC	Mand.	MRND ipsi.	IIc	2	V	1	1	Swallow-ing	4	< 60 Gy	n
21	M	56.8	OSCC	Mand.	SOND ipsi.	II	2	V	19	1			< 60 Gy	
22	M	51.1	OSCC	Mand.	MRND ipsi.	II	2	V	12	2			< 60 Gy	
23	M	70.6	OSCC	Mand.	SOND ipsi.	III	3	V	5	1			≥ 60 Gy	
24	M	24.4	OKC	Max.	None	II	2	V	5	4	Swallow-ing	6	-	y (11 mo)
25	M	14.8	OS	Max.	None	III	1	V	1	1	Trismus	4	< 60 Gy	n
26	M	40.8	OM	Mand.	None	IV	3	V	5	2			-	
27	F	65.1	BPONJ	Mand.	None	III	3	V	8	2	Bony masses	8	-	n
28	M	30.0	ORN	Mand.	None	I	1	A	21	2			≥ 60 Gy	

29	F	40.3	OSCC	Mand.	None	III	1	A	50	1		< 60 Gy

ACC, adenoid cystic carcinoma; ACIN, Acinus cell carcinoma; AMB, Ameloblastoma; BPONJ, bisphosphonate related necrosis of the jaw; OKC, odontogenic keratocyst; OM, Osteomyelitis; ORN, Osteoradionecrosis; OS, Osteosarcoma; OSCC, Oral squamous cell cancer; MR, modified radical, SO, supraomohyoidal; ND, neck dissection.
